# Improving swallowing outcomes in patients with head and neck cancer using a theory-based pretreatment swallowing intervention package: protocol for a randomised feasibility study

**DOI:** 10.1136/bmjopen-2016-014167

**Published:** 2017-03-27

**Authors:** Roganie Govender, Christina H Smith, Benjamin Gardner, Helen Barratt, Stuart A Taylor

**Affiliations:** 1 University College London Hospital, Head & Neck Cancer Centre, London, UK; 2 Department of Behavioural Science & Health, University College London, London, UK; 3 Division of Psychology & Language Sciences, University College London, London, UK; 4 Department of Psychology, Institute of Psychiatry, Psychology and Neuroscience (IoPPN), Kings College London, London, UK; 5 Department of Applied Health Research, NIHR CLAHRC North Thames, University College London, London, UK; 6 Centre for Medical Imaging, University College London, London, UK

**Keywords:** dysphagia, swallowing exercises, feasibility study, head and neck cancer, behaviour change techniques

## Abstract

**Introduction:**

The incidence of head and neck cancer (HNC) in the UK is rising, with an average of 31 people diagnosed daily. Patients affected by HNC suffer significant short-term and long-term post-treatment morbidity as a result of dysphagia, which affects daily functioning and quality of life (QOL). Pretreatment swallowing exercises may provide additional benefit over standard rehabilitation in managing dysphagia after primary HNC treatments, but uncertainty about their effectiveness persists. This study was preceded by an intervention development phase to produce an optimised swallowing intervention package (SIP). The aim of the current study is to assess the feasibility of this new intervention and research processes within a National Health Service (NHS) setting.

**Method and analysis:**

A two-arm non-blinded randomised controlled feasibility study will be carried out at one tertiary referral NHS centre providing specialist services in HNC. Patients newly diagnosed with stage III and IV disease undergoing planned surgery and/or chemoradiation treatments will be eligible. The SIP will be delivered pre treatment, and a range of swallowing-related and QOL measures will be collected at baseline, 1, 3 and 6 months post-treatment. Outcomes will test the feasibility of a future randomised controlled trial (RCT), detailing rate of recruitment and patient acceptance to participation and randomisation. Salient information relating to protocol implementation will be collated and study material such as the case report form will be tested. A range of candidate outcome measures will be examined for suitability in a larger RCT.

**Ethics and dissemination:**

Ethical approval was obtained from an NHS Research Ethics Committee. Findings will be published open access in a peer-reviewed journal, and presented at relevant conferences and research meetings.

**Trial registration number:**

ISRCTN40215425; Pre-results.

**Strengths and limitations of this study**

- Use of a randomised controlled trial design to minimise bias and differences between groups.
- Study design incorporates prior qualitative work to optimise adherence to the intervention.
- Method includes consultation with clinicians involved in usual care to devise a usual care protocol to facilitate consistency.
- Limited to one hospital site.
- Patients and clinicians are not blinded to randomisation allocation.

## Introduction

### Background

Incidence of head and neck cancer (HNC) in the UK is rising^1^ with an average of 31 people receiving a new diagnosis daily.^2^ This increase is primarily attributed to the rise in human papilloma virus (HPV)-related oropharyngeal cancer.^3^ Individuals with HPV-induced malignancy are often much younger than those with cancers induced by smoking and alcohol, and often otherwise healthy and in active employment. Reducing the morbidity of cancer treatments is a priority for the NHS Cancer Reform Strategy^4^ and the Macmillan Living with and Beyond Cancer Programme.^5^ Patients affected by HNC suffer significant short-term and long-term post-treatment morbidity as a result of dysphagia, which affects daily functioning and quality of life (QOL). Swallowing difficulties may arise secondary to the presence of tumours in the mouth and throat, 6 from damage or resection of swallowing-related soft tissues and nerves during surgical intervention^7^ and from the side effects and long-term tissue damage following chemoradiation.^8^ Swallowing interventions that may ameliorate the problems associated with eating and drinking after primary HNC treatments (surgery, chemoradiotherapy) are an important part of the care delivered to patients diagnosed with HNC.^9^

### Current UK practice for managing dysphagia in HNC

Traditionally, patients would have seen a speech and language therapist (SLT) following their cancer treatment for rehabilitation of their swallowing and communication difficulties. A gradual shift in practice occurred following publication of the National Institute for Health and Care Excellence (NICE) Improving Outcomes Guidance,^10^ which recommend that patients should also see an SLT *prior* to their treatment to inform them of the *likely* impact to their swallowing and speech function. This information giving is also viewed as a necessary part of the process of informed consent. In support, an emerging number of functional outcome studies have indicated that swallow function pre treatment could be a strong predictor of long-term swallow function post-treatment.^11–13^

Implementation of the NICE recommendation is currently variable. In some centres, patients are provided with brief information by the SLT at the time of seeing their medical consultant in a multidisciplinary clinic setting. In other centres, separate SLT consultations take place, which include documentation of baseline functional measures for swallowing and communication, as well as advice on diet modification and the recommendation to start a general protocol of swallowing exercises. The latest report from the UK National Database of Head and Neck Oncology (DAHNO)^14^ indicated that only 29% of patients with HNC in England and Wales were recorded as having a pretreatment SLT consultation. Given the clinical and financial resource implications of a separate pretreatment SLT consultation, implementation will likely only increase if there is clear specification as to the optimal content of the consultation, together with evidence supporting its benefit on patients' outcomes. Aside from information giving, there is a need to define what interventions administered *before* treatment (prehabilitation) may improve patient experience and/or post-treatment function.

### The role and context of pretreatment swallowing exercises

**Figure 1:**
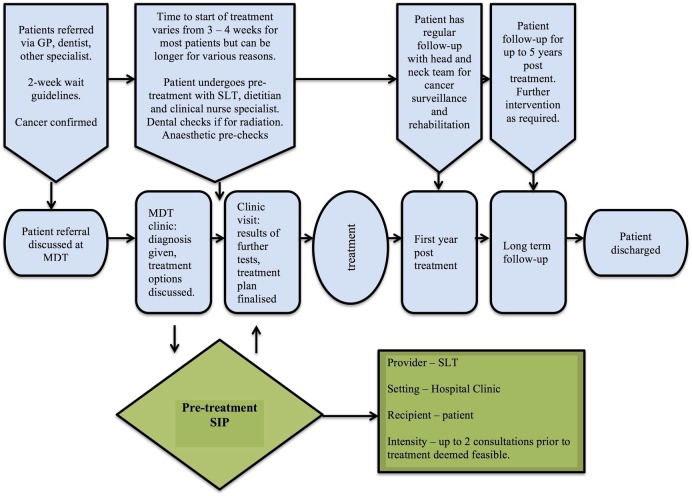
Pathway of care for patients with head and neck cancer.

The physiological rationale for prophylactic swallowing exercises has been previously described in the literature. Strength-based exercises and/or range of movement exercises aimed at the swallowing musculature may prevent muscle atrophy and reduce or delay the impact of radiation-induced fibrosis.^15–18^ Preconditioning through exercises has been reported to be helpful in other types of surgery.^19^ Swallowing is described as a ‘suboptimal activity’^20^ meaning that it can be adequate for the purpose of obtaining oral nutrition even when not at maximal physiological functioning. While it seems intuitive that pretreatment swallowing exercises should be helpful in increasing physiological reserve, reducing disuse atrophy and possibly delaying the onset of fibrosis, uncertainty about its effectiveness in improving swallowing for patients with HNC persists.^21^ Few randomised clinical trials have attempted to address this knowledge gap, with mixed results.^17^ ^22–28^ Trials evaluating the effects of pretreatment swallowing exercises suffer limitations in study design, and the lack of consistent outcome measures across studies is problematic.^21^ Uncertainty also remains as to the optimal type and frequency of exercises although such considerations are less relevant to tailored interventions guided by prior physiological assessment. The practice of providing prophylactic swallowing exercises varies among UK clinicians,^29^ and to date there is no published UK data supporting the effectiveness of pretreatment swallowing exercise interventions in the HNC population.^21^ Any pretreatment intervention package will need to address often poor patient adherence to swallowing exercises.^22^ ^24^ ^30^ Improved adherence may be achieved by facilitating a change in patient behaviour. A new intervention will also need to be compatible with the broader cancer care pathway as detailed in figure 1.

### Development of a tailored pretreatment swallowing intervention package (SIP)

In preparation for the feasibility study described in this manuscript, an optimised SIP was developed by the researchers, guided by the methodology proposed by the Medical Research Council (MRC) for devising complex interventions incorporating multiple components that may interact in producing outcomes.^31^ The scope of the current work relates to the development and feasibility testing phases of a complex intervention, but the framework also includes evaluation and implementation phases with updated guidance that incorporates a *process* evaluation.^32^ We briefly describe the development work below.

Recognising the poor patient adherence reported in previous studies of swallowing exercises,^22^ ^24^ ^30^ we paid specific attention to optimising patient adherence during the development phase by drawing on theoretical frameworks and tools (The Behaviour Change Wheel, Behaviour Change Technique Taxonomy v1) from the field of behavioural science.^33^ ^34^

The new SIP—Swallowing Intervention Package: Self-Monitoring, Assessment, Rehabilitation Training (SIP SMART) was devised using current best evidence of swallowing assessment,^35^ as well as insights from our earlier studies exploring the behavioural dimensions of this complex intervention; a systematic review of the literature^36^ and a patient interview study *(submitted manuscript).*

In our systematic review of behavioural swallowing intervention studies,^36^ we used a published taxonomy (BCTTv1)^33^ of 93 hierarchically organised behaviour change techniques (BCTs), to identify the BCTs reported in the literature. BCTs may be defined as the smallest active ingredient of an intervention that may bring about a change in behaviour, for example, *demonstration of the behaviour* or *self-monitoring of the behaviour*. We surmised that the BCTs that were more frequently associated with successful interventions were more likely to be successful in future interventions.

We also carried out semistructured interviews with a group of patients (n=13) who had completed treatment for HNC to obtain their views and experiences of swallowing rehabilitation exercises, and in addition obtained feedback from respondents about the potential use of video animations (Dysphagia App, Northern Speech Services, USA) as an educational tool in explaining the basic mechanics of swallowing. The barriers and facilitators to exercise adherence revealed in our qualitative study informed our behavioural analysis and the subsequent selection of BCTs for the SIP SMART intervention.

**Figure 2:**
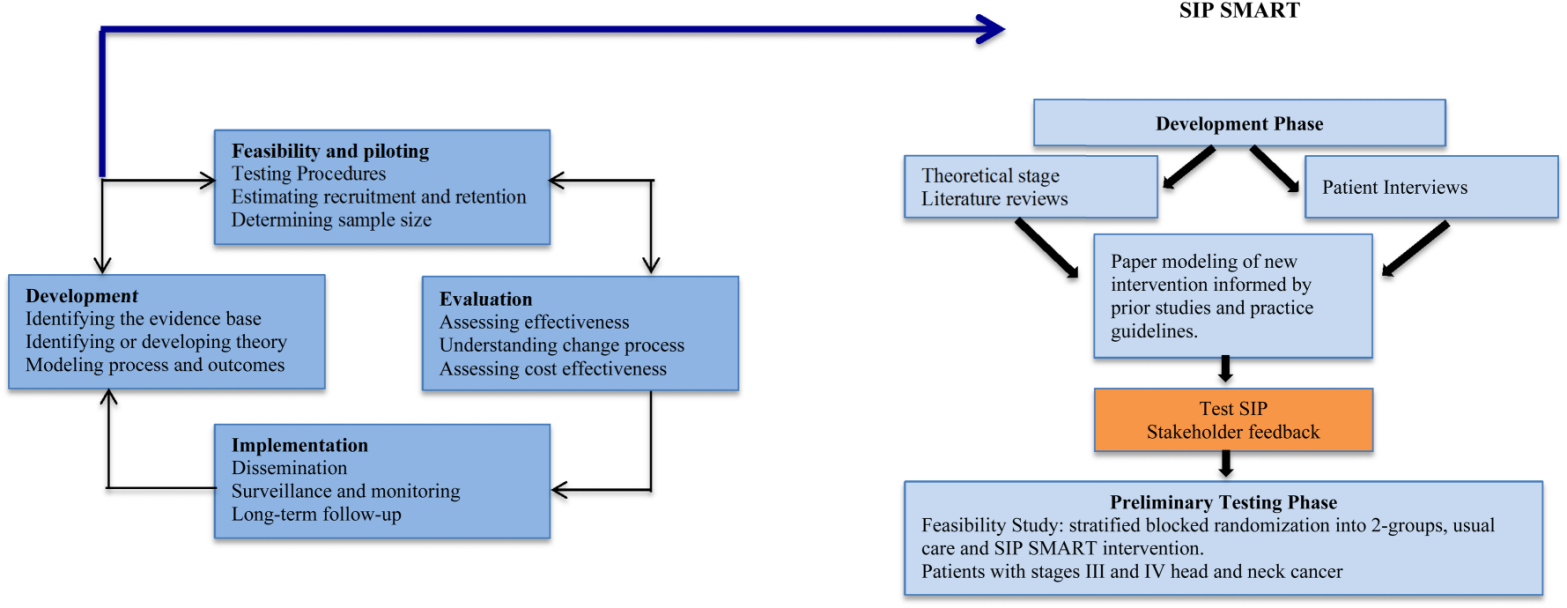
Key stages of the *MRC complex interventions framework that informed the development and preliminary testing of the SIP SMART intervention. *Source: Craig *et al*, BMJ;2008. MRC, Medical Research Council; SIP SMART, Swallowing Intervention Package: Self-Monitoring, Assessment, Rehabilitation Training.

We ensured that the new SIP would meet mandatory guidelines^10^ for information provision and informed consent, and that it could feasibly be incorporated within the existing cancer pathway for patients with HNC. The new SIP was discussed and refined by clinician (Royal College of Speech and Language Therapist expert clinician group (RCSLT)) and patient (patient–public involvement group (PPI)) stakeholder groups. Intervention manuals were produced for the new intervention and usual care in collaboration with the stakeholder groups. Figure 2 is a schematic diagram illustrating an overview of this process and how it links to the MRC complex interventions framework.

Although another complementary non-randomised UK trial of pretreatment swallowing intervention is planned,^37^ our SIP SMART trial is unique as it is based on an optimised pretreatment intervention using tailored exercises selected after physiological assessment of swallowing, and is directly informed by theoretically derived BCTs. Furthermore, comprehensive evaluation of a new SIP is likely best performed using a randomised controlled trial (RCT) design. Given the uniqueness of our SIP and proposed future RCT evaluation, a feasibility study was deemed imperative to ascertain viability within a NHS context, and to identify and address potential barriers to future roll out on a larger scale.

In particular, the aims of this feasibility study are as follows:

- Assess the rate of recruitment of eligible participants and identify any specific barriers to recruitment.
- Determine the acceptability of randomisation, and the randomisation procedure to patients and the clinical care team.
- Determine retention and attrition over the time course of the study.
- Evaluate the ease of protocol implementation, including research processes, and identify barriers in the clinical setting.
- Evaluate a range of potential outcome measures, including the ease and completeness of data collection across various time points.
- Determine concordance between potential outcome measures and define the most suitable primary outcome for the definitive study.
- Collect data to inform future sample size calculation.

## Methods and analysis

### Study design

This study forms the preliminary testing phase described within the MRC complex interventions framework. Following on from our development work, we will conduct a two-arm parallel group non-blinded randomised controlled feasibility study. The SPIRIT^38^ (Standard Protocol Items for Randomised Interventional Trials) checklist of was used to inform the content of this protocol.

### Study population, setting and recruitment plan

The study will take place at a single NHS hospital site (Head and Neck Cancer Centre) with a catchment population of 1.5 million. The study sample will be drawn from the population of patients with newly diagnosed HNC referred to the cancer centre, and discussed at the weekly multidisciplinary team (MDT) meeting. Potential patients for the study (based on diagnosis) will be identified during the meeting by the researcher (RG), research nurse or other members of the MDT. The research team will ensure that the treating consultant is aware of potentially eligible patients so that he/she may introduce the study during the consultation with the patient if appropriate. For this feasibility study, sample size was determined pragmatically using the general guidance suggested by Lancaster and colleagues^39^ who recommend that n of 30 is sufficient to estimate key parameters in a feasibility study. Based on a conservative annual referral of ∼70 newly diagnosed stage III and stage IV patients with HNC to the Head and Neck Centre, we estimated that it will take about 9 months to recruit a total of 32 patients to this study based on recruiting 60% of eligible patients. Eligibility criteria for inclusion in the study are listed in box 1.

Box 1 **Inclusion and Exclusion Criteria**

Inclusion:

- Patients with newly diagnosed stage III and stage IV head and neck cancer.
- Discussed at the head and neck MDT and planned for treatment via surgery and/or chemoradiotherapy or combinations thereof.
- Able to provide informed consent.
- Proficiency in English satisfactory to participate/engage in the intervention.
- Aged 18 and above.

Exclusion:

- Patients with previous head and neck cancer treatment.
- Patients who are mid treatment or those receiving palliation.
- Patients who are to be treated solely by non-standard treatment that is not surgery, radiotherapy, chemoradiotherapy or combinations thereof. Patients treated by chemotherapy, brachytherapy and photodynamic therapy alone will be ineligible.
- Patients who are planned for a total laryngectomy.
- Patients who are considered vulnerable or unable to provide informed consent.
- Patients with brain tumours and other primary sites not within head and neck.

### Prescreening/screening

All patients who meet the clinical eligibility criteria identified at each MDT meeting will be recorded on the study screening log by the researcher or research nurse. Screening will take place at the outpatient clinic consultation when treatment options are discussed by the surgeon/oncologist. The researcher/clinician will attend the consultations for eligible patients. If appropriate at this stage, the purpose of the study will be explained and patients will be given the patient information leaflet to take away. Most patients will be booked for repeat visits to the head and neck clinic prior to finalising their treatment plan. Due consideration will be given to ensuring that the study information is discussed at an appropriate time after the diagnosis. Patients will be given a minimum of 24 hours after receiving the patient information sheet before a mutually agreed time is arranged to answer any further questions to assist patients in deciding about whether to participate. The time frame was chosen because most patients return to the hospital for other tests the day after their clinic visit. This offers an opportunity to answer questions in person and obtain signed consent if appropriate. Patients will be reassured that participation is voluntary with the freedom to withdraw at any stage, and that participation in the study will not affect or delay their main treatment.

### Enrolment/consent

Informed consent will be obtained by the researcher/clinician (RG) or a trained research nurse. Following informed consent and generation of a patient study identification number, the patient will be entered onto the study enrolment log and randomised to either the SIP SMART intervention or usual care group as detailed below.

### Randomisation and allocation

**Figure 3:**
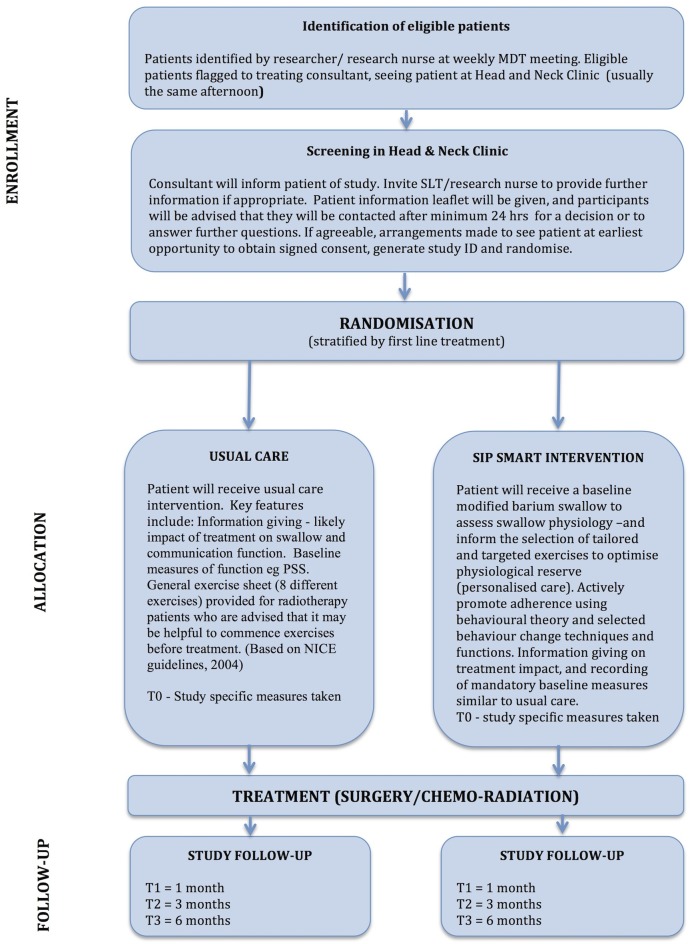
Trial flow chart.

Eligible patients will be randomly assigned in a 1:1 ratio between usual care and intervention groups (figure 3 for trial flow chart). Patients will be stratified by first-line treatment; surgery or chemoradiation, a known factor that impacts swallowing outcomes.^13^ It will therefore be necessary to ensure a balance of primary treatment modality across the groups. Owing to the small numbers in this study, random block permutations will be employed to ensure a close match in numbers in the intervention and usual care groups at any given point during the trial.

Patients will be allocated to one of two groups using an online computer-generated randomisation service provided by an external company: http://www.sealedenvelope.com/. The company is registered with the Information Commissioners Office (ICO) and inspected by the MHRA (UK trials regulator). Following consent, the researcher or research nurse will enter the password-protected website and complete relevant information regarding first-line treatment. Randomisation is immediate, and the group allocation is emailed within a few minutes. This process is undertaken in the presence of the patient after signed consent is obtained, ensuring that the allocation is concealed until this point and simultaneously made known to the patient and researcher. Allocation is not blinded as the patient and staff will be aware that the new intervention includes a baseline videofluoroscopy or X-ray of swallowing. Patients allocated to the usual care group are advised that they will be sent an appointment in the post to see an SLT prior to their treatment as per the usual care pathway. Patients allocated to the intervention group are given an appointment and a further information leaflet on having a videofluoroscopy to assess how well the muscles and nerves function when swallowing different textures of food and drink. All patients are given three questionnaires to complete and return prior to their appointment with the SLT.

### Interventions and procedures

*Usual care group*: This group will receive the usual pretreatment care offered by the SLT prior to their upcoming surgery and/or chemoradiation. The SLT clinical team consisting of four members participated in a series of consensus meetings regarding the delivery of usual care to facilitate equipoise. All four team members involved in the delivery of usual care have also undergone good clinical practice (GCP) training. A usual care manual was written and agreed by the SLT clinical team prior to initiation of the study to ensure a level of consistency among the clinicians. Usual care pretreatment is one 45 min consultation as described below:

- General case history taking and introduction of SLT role.
- Clinical baseline screening of swallowing and communication function. This is usually based on an oro-motor assessment; 100 mL water swallow test;^40^ a clinician-rated Performance Status Scale indicating the normalcy of diet texture and public eating score;^41^ and a clinician-rated scale for chewing, communication and swallowing.^42^ Maximal jaw opening using a TheraBite measure and voice quality ratings using the GRBAS (Grade of Hoarseness, Roughness, Breathiness, Asthenia, Strain) rating scale are also recorded.
- The patient is provided with a general overview of the planned treatment (surgery or chemoradiation) and information about the likely side effects such as mucositis and taste changes and impact of treatment on swallowing and communication function.
- General advice and exercises are offered to patients planned for chemoradiation at this appointment. Patients are provided with a general exercise sheet that includes instructions for eight different swallowing exercises, for example, passive jaw stretches. This is included as part of the information pack given to all patients receiving radiotherapy. Patients are advised that it *may* be helpful to start doing the exercises before treatment.

*Intervention group*: Patients in the intervention group will be pretreated according to the SIP SMART protocol that includes the specific components of the new intervention in addition to all aspects of usual care. The intervention takes place over two 45 min consultations that may follow each other on the same day or with a day or two between them depending on patient preference. The new intervention will be delivered by one clinician (RG) who completed a 5-day intensive training course in behaviour change (UCL Centre for Behaviour Change), supplemented by online training in coding BCTs, as well as ongoing mentorship from an expert in behaviour change. Specific details of the new intervention have not been explicitly shared with clinicians delivering usual care to minimise contamination. Broad differences include the following:

- Patients will undergo a radiological assessment of their swallow function in the fluoroscopy suite at the hospital site. This procedure is part of the SIP SMART intervention informing the selection of targeted exercises, and is used as part of patient education. A standard protocol for this clinical procedure will be adopted for the study.^35^ The patient will be asked to swallow a variety of food textures; fluoroscopic images of the lateral and anterior–posterior plane captured at a rate of 30 frames per second will be recorded on a swallow workstation (Digital Kay Pentax Swallow workstation, USA). This will be available for later analysis of swallow physiology. The fluoroscopy screening time is usually 2–3 min.
- Patients will subsequently be shown a video animation (Dysphagia App, Northern Speech Services, USA) to explain the basic mechanics of swallowing and to orientate them to key structures such as the tongue, base of tongue, airway and oesophagus. Patients will thereafter be shown their own videofluoroscopy and helped to identify the key structures using this newly acquired knowledge. The clinician will encourage the patient to provide commentary and or ask questions as they watch their own swallowing.
- The videofluoroscopy assessment will be used to tailor the information, advice and exercises given to the patient during the pretreatment session and to facilitate discussion about the rationale for exercises and possible consequences of not doing exercises.
- Further detail about the intervention content and behavioural strategies used is provided in the SIP SMART manual. This is not included in this paper, but can be requested from the first author.

Patients in both groups will follow the usual care pathway for SLT rehabilitation post treatment (see figure 3 for trial flow chart). The number of SLT rehabilitation sessions for all patients will be recorded. Patients will be informed that exercises may be amended post treatment based on updated swallowing assessment.

### Baseline and follow-up outcome measures

Swallowing is a multidimensional phenomenon that may be measured by a number of different indicators including: patient-reported outcome measures, clinician ratings and scores from instrumental assessments such as videofluoroscopy and fibre-optic endoscopic evaluation of swallowing. This range of outcomes can prove problematic when synthesising findings from multiple studies. In this feasibility trial, we have selected a similar panel of swallowing outcome measures to that used in concurrent UK trials for HNC (radiation and surgery-based trials using a swallowing outcome measure),^43^ as well as measures collected as part of routine clinical practice (table 1). Outcome measures will be collected at baseline and 1, 3 and 6 months after treatment. Six months represents a relatively stable time point in the recovery trajectory for patients with HNC and was therefore selected as an appropriate end point.^21^ ^36^ Patient weight, body mass index and use of feeding tube will also be recorded at all time intervals. The MD Anderson Dysphagia Inventory (MDADI)^44^ will be used to determine swallowing related QOL. We have chosen to collect information on health-related QOL via the Functional Assessment of Cancer Therapy and Head and Neck subscale (FACT-HN) as this questionnaire was identified as the most preferred by patients with HNC when compared with other QOL questionnaires.^45^

**Table 1:** Outcome measures and time points

Measure	T0 baseline	T1 1 month	T2 3 months	T3 6 months
Background information	X			
Measures taken as part of usual care				
Performance Status Scale (PSS)	X	X	X	X
Maximal incisor opening (mouth opening)	X	X	X	X
Functional Intraoral Glasgow Scale	X	X	X	X
100 mL Water swallow test	X	X	X	X
Additional measures for trial				
MD Anderson Dysphagia Inventory	X	X	X	X
General Self-Efficacy Scale	X			X
Self-reported adherence question		X	X	X
HRQOL—FACT–HN	X	X	X	X
Modified Barium Swallow Impairment Score and Penetration/Aspiration score				X
Acceptability to participation/randomisation questionnaire				X

HRQOL—FACT–HN, Health Related Quality of Life- Functional Assessment of Cancer Treatment- Head & Neck subscale.

At 6 months, a videofluoroscopy will be conducted on all patients using the standardised Modified Barium Swallow Impairment Profile Protocol (MBS Imp Profile), and analysis.^35^ Three SLT clinicians who have completed a 25 hour online training module and obtained the minimum 80% reliability score will provide consensus ratings for these assessments. Standard assessment rating forms developed as part of the MBS Imp Profile will be used to score the videofluoroscopies. We will also rate aspiration from the videofluoroscopies, based on the widely used 8-point Penetration-Aspiration Scale (PAS).^46^

As we anticipate that adherence to swallowing exercises should be associated with better swallowing, we will also collect intermediate outcomes on adherence using two simple questions developed for this trial. The questions will ask about percentage adherence over a specified time, and a free text question to gather further information about any specific reasons for adherence/non-adherence to exercises.

### Safety considerations

We do not anticipate any serious safety concerns arising from this largely behavioural intervention. The use of the videofluoroscopy as part of the intervention is sometimes associated with aspiration of barium contrast material but barium pneumonitis is reported to be rare at <1%.^47^ The additional radiation exposure associated with the procedure is roughly the equivalent of 2 months of background radiation that an adult in the UK may experience from the environment, and considered minor in the context of the patient's overall treatment. The procedure will be undertaken by an experienced SLT familiar with the protocol for dealing with an adverse event related to barium inhalation. The procedure is also a well-established part of routine clinical practice.

### Data collection and management

We devised a number of study specific forms including a case report form (CRF), screening log, enrolment log, training log for any study-related training and file note entry forms for any relevant ad hoc communication. Patient names will be replaced by a study number on all study forms and completion of CRFs will be in accordance with GCP guidelines. A site file containing all relevant documents as stipulated by local research and development and governance guidelines will be maintained throughout the study and securely stored in a locked cabinet. All patient-reported questionnaires will also be securely filed. Non-identifiable quantitative data will be transferred from the questionnaires and CRFs to a specifically designed Microsoft Access database by a data manager. The database will first be tested using mock data to ensure that it meets the requirements for data entry. A random check of ∼10% of the data inputted will be reviewed against the original source by the researcher (RG) to estimate an error rate. This information will help ensure that a robust data management plan is in place for a more formal trial. The researcher will maintain an electronic diary of relevant information pertaining to the study processes on a password-protected laptop computer.

### Analysis

We will use mainly descriptive analysis and summary statistics to address our aims. Study screening and enrolment logs will be used in determining the rate of recruitment into the study. All qualitative information (researcher diary), minutes of study-related discussions and meetings will be imported into NVIVO 10, a software database to facilitate the organisation and thematic coding of qualitative or textual data. The researcher is in a unique position of being embedded within the clinical team, and therefore able to make observations over the duration of the study in a naturalistic manner. This approach to process analysis arguably may provide more useful information than the use of post hoc focus groups and interviews that rely on participant memory and may be removed from context.^48^ Observing and collecting information in this way also means that the researcher can observe the interplay of other factors (eg, multiple studies competing for the same patient group, prevailing views of the treating consultant about the value of the proposed intervention and how busy the clinic is) that may reveal vital information about the barriers and facilitators to recruitment.

We will also report on the practicality of implementing the protocol, for example, obtaining timeslots for videofluoroscopy; average time taken for the recruitment and consent process; utility of the chosen randomisation method and suitability of the study-specific forms including the CRF. This information will be used to optimise components of the protocol and study process in preparation for a larger trial.

As a range of outcome measures will be collected, we will look at the suitability of each measure, the ease of collection and the quality and completeness of the data collection. We will observe the relationships (concordance and discordance) between the different outcome measures to help inform the most suitable choice of primary and secondary measures for a definitive trial. Important parameters such as SD and estimates of effect size will be used to inform the sample size calculation. Based on the available literature, we will aim to specify the target difference (clinically meaningful difference) for the chosen primary outcome. The primary outcome measure will be chosen from potential candidate measures on the basis that it is valid, practical and feasible to obtain and has expert agreement (RCSLT clinical expert group) that it reflects a good summary measure to answer the question of whether the new intervention is effective in improving swallowing.

Patient acceptability to participation and randomisation will be determined using a previously developed questionnaire.^49^ Self-reported adherence to the intervention will be explored via a brief study questionnaire. Previous studies reported that full adherence to exercises during radiotherapy was under 15%.^27^ ^30^ In a Danish study of a similar intervention to the current study, an average of 35% of patients reported doing their exercises at least once a day between 1 month and 11 months after treatment.^22^ We have therefore selected 35% as the minimum target adherence for our study.

### Criteria for success

This study will be viewed as feasible to proceed to a definitive trial if:

- a suitable outcome measure is determined, and sample size estimated;
- recruitment rate into the trial reaches an average of four patients a month;
- patients report generally positive views about participation and acceptance to randomisation as determined by questionnaire evaluation;
- patients in the intervention group are more adherent than those in usual care, with at least 35% of intervention group patients reporting satisfactory to good adherence to exercises.

## Discussion

To the best of our knowledge, this is the first randomised UK study of a behavioural swallowing exercise intervention for patients with HNC registered on the trials database. By undertaking a feasibility study and identifying key uncertainties, any future study may be optimised to make best use of resources.^50^ This study follows earlier work on intervention development bringing together expertise from different fields including clinical dysphagia management and behaviour change. It benefits from the use of newer paradigms in health research including the use of consultative and consensus meetings to devise a treatment manual to specify the content of *usual care*, a common omission when reporting such interventions.^51^ It is likely to provide a rich source of information about how readily patients with a new diagnosis of HNC will accept and participate in a self-management-type intervention. It will also provide a preliminary indication of the recruitment potential for rehabilitation therapy trials for this population. A recent UK study^52^ that randomised HNC patients to either a pretreatment gastrostomy tube or a nasogastric tube reported recruiting only 23% of eligible patients, highlighting the importance of this feasibility work. It is well known that the treatment of HNC involves a complex care pathway with multiple disciplines being involved, particularly at the pretreatment stage. The feasibility of undertaking a clinical trial at this point in the patient pathway is compounded by the challenges of approaching patients to participate in a trial shortly after receiving a cancer diagnosis. Insights from this study may therefore have more widespread relevance for future studies of this population.

Limitations of the study include the inability to blind participants and staff to the randomisation allocation as all patients in the intervention group will receive a videofluoroscopy as part of the intervention. Based on the limited number of individuals trained in rating videofluoroscopies using the MBS impairment profile, the same SLTs involved in usual care delivery will be rating the assessments. For this reason, we have chosen to use a consensus rating from three clinicians as this method is likely to introduce the least bias. We have not planned to evaluate fidelity in delivering the intervention at this stage, as only one individual will be delivering the intervention (RG). In a larger trial, training will be required by all clinicians before delivering the intervention and fidelity checks will be built into the research process. In spite of these limitations, the current study represents a first and important step towards examining the feasibility of undertaking a full-scale RCT within NHS hospitals to determine the effectiveness of a pretreatment swallowing intervention for patients with HNC, delivered by SLTs.
